# Green synthesis of bimetallic ZnO–CuO nanoparticles and their cytotoxicity properties

**DOI:** 10.1038/s41598-021-02937-1

**Published:** 2021-12-06

**Authors:** Yan Cao, Hayder A. Dhahad, M. A. El-Shorbagy, Hajar Q. Alijani, Mana Zakeri, Abolfazl Heydari, Ehsan Bahonar, Miroslav Slouf, Mehrdad Khatami, Mahin Naderifar, Siavash Iravani, Sanaz Khatami, Farnaz Farzaneh Dehkordi

**Affiliations:** 1 School of Mechatronic Engineering, Xi’an Technological University, Xi’an, 710021 China; 2 Mechanical Engineering Department, University of Technology, Baghdad, Iraq; 3 Department of Mathematics, College of Science and Humanities in Al-Kharj, Prince Sattam Bin Abdulaziz University, Al-Kharj, 11942 Saudi Arabia; 4 Department of Basic Engineering Science, Faculty of Engineering, Menoufia University, Shebin El-Kom, 32511 Egypt; 5 Department of Biotechnology, Shahid Bahonar University of Kerman, Kerman, Iran; 6 Department of Biology, Islamic Azad University, Tehran Medical Branch, Tehran, Iran; 7 Polymer Institute of the Slovak Academy of Sciences, Dúbravská Cesta 9, 845 41 Bratislava, Slovakia; 8 Faculty of Chemical and Petroleum Engineering, Sahand University of Technology, Tabriz, Iran; 9 Institute of Macromolecular Chemistry, Czech Academy of Sciences, Heyrovskeho nam. 2, 162 06 Prague 6, Czech Republic; 10 Noncommunicable Diseases Research Center, Bam University of Medical Sciences, Bam, Iran; 11 Faculty of Nursing & Midwifery, Zabol University of Medical Sciences, Zabol, Iran; 12 Faculty of Pharmacy and Pharmaceutical Sciences, Isfahan University of Medical Sciences, Isfahan, Iran; 13 Department of Medical Biotechnology, School of Advanced Technologies in Medicine, Shahid Beheshti University of Medical Sciences, Tehran, Iran; 14 Department of Biology, Ardabil Branch, Islamic Azad University, Ardabil, Iran

**Keywords:** Biological techniques, Biotechnology, Chemical biology

## Abstract

In this study, a simple and green strategy was reported to prepare bimetallic nanoparticles (NPs) by the combination of zinc oxide (ZnO) and copper oxide (CuO) using *Sambucus nigra* L. extract. The physicochemical properties of these NPs such as crystal structure, size, and morphology were studied by X-ray diffraction (XRD), field emission gun scanning electron microscopy (FEG-SEM), and transmission electron microscopy (TEM). The results suggested that these NPs contained polygonal ZnO NPs with hexagonal phase and spherical CuO NPs with monoclinic phase. The anticancer activity of the prepared bimetallic NPs was evaluated against lung and human melanoma cell lines based on MTT assay. As a result, the bimetallic ZnO/CuO NPs exhibited high toxicity on melanoma cancer cells while their toxicity on lung cancer cells was low.

## Introduction

Cancer can cause the unregulated cell growth with high potential to invade and spread to other cells and tissues of the body by the lymph system and blood via a metastatic process^1,2^. Cancer as one of the most aggressive diseases kills more than ten million people every year^3^. Lung cancer and melanoma are the most deadly ones. One of the most important features of the cancer cells compared to somatic cells is their ability to replicate and spread to different parts of the body. Cancer cells release a variety of factors into their environment which can change the function of cells in the tumor microenvironment^4^. One of the reasons for the high mortality rate of patients with lung cancer and melanoma is the uncontrolled growth of cancer cells in the lung and skin tissue, metastasis and spread to sensitive organs (such as the brain). Currently, the most common treatments for cancer are surgery, radiation therapy, immunotherapy, hormonal therapy, and chemotherapy^5^, but recently many researchers have turned to nanomaterials and herbal anticancer drugs^6,7^. Despite some advantages, conventional chemotherapy is discouraging to invest more in drug discovery and/or drug delivery systems due to some drawbacks such as poor bioavailability of drugs to tumor tissues, adverse side effects, low therapeutic indices, high dose requirements, non-specific targeting, and multidrug resistance^8,9^. Different evaluations have been performed on the anticancer properties of various nanoscaled structures, composites, RNAs, and polymers^10–23^.

The biomedical potentials of various secondary metabolites such as phenolic compounds, flavonoids, glycosides, terpenoids and aldehydes have been evaluated^24–26^. Therapeutic methods based on the application of encapsulated herbal compounds in nanomaterials^27^, hyperthermia, targeted delivery of genes or anticancer drugs to the cells/tissues have resulted in efficient cell death of cancer cells in the targets^28^. Indeed, rapid proliferation of cancer cells and necrosis of normal cells in the body are prominent features of cancer cells and side effects of current cancer therapy. Rapid and targeted penetration in cancer cells can prevent the spread of disease to other tissues, and thus control the cancer. Nanoparticles (NPs) have gained interest of scientists in the field of nanomedicine^29–36^. The therapeutic effects of NPs depend on the particle size, the culture time of the target cell, the amount of metal in the targeted cell, and their physicochemical properties^36–39^. On the other hand, bimetallic and multimetallic NPs have shown unique physicochemical properties with synergistic effects and high functionality^40^. These integrated NPs have more reactive sites, increased efficiency, and greater stability^41,42^. In recent decades, the antitumor activity of different biogenic and non-biogenic bimetallic NPs has been evaluated^43–45^. In 2019, Lomelí-Marroquín et al*.* synthesized silver/gold bimetallic NPs with the aim of reducing the nano metallic toxicity of silver, and evaluated their antitumor activity on melanoma cancer cells^46^. Pt/Pd bimetallic NPs were synthesized using *Dioscorea bulbifera* extract, and gold-silver bimetallic NPs were synthesized using *Stigmaphyllon ovatum* leaf extract; their anticancer activity against HeLa cells was also investigated^47,48^. In another study, the gold(i)-BODIPY-imidazole bimetallic complex demonstrated good anti-proliferative activity against breast, colon, and prostate cancer^49^. In 2019, silver/palladium bimetallic NPs were synthesized using *Terminalia chebula* fruit extract, and their antitumor properties were evaluated against A549^50^. Additionally, biogenic copper and zinc oxide NPs were synthesized in 2018 and 2019, and their anticancer activities were evaluated against T98G human glomerular superficial cell and cervical cancer, respectively^51,52^. Typically, the synthesis of multimetallic and bimetallic NPs is costly and time-consuming, and may include toxic or hazardous substances. Metal oxide NPs such as zinc oxide (ZnO) and copper oxide (CuO) demonstrated diverse biological applications; these NPs were highly compatible with normal cells in the body. Biogenic ZnO NPs were eco-friendly synthesized using *Cardiospermum halicacabum* and *Mangifera indica* leaf extracts, and they illustrated good antitumor properties against A375 and A549 cells at a concentration of 50 μg/mL, respectively^4,53^. In another study, CuO NPs were synthesized using a green method; these NPs inhibited mRNAII expression in A549 cancer cells and stimulated apoptosis^54^. In 2017, Chakraborty et al*.* reported an IC_50_ (the concentration of NPs caused 50% mortality) of 1.71 μg/mL for CuO NPs against A375 cancer cells^55^.

ZnO and CuO NPs can be applied in cancer therapy, cosmetic creams, and industrial catalysts. ZnO NPs are widely employed due to the production of reactive oxygen species (ROS) and their unique electrostatic behavior, which can prevent DNA damage. At physiological pH, ZnO NPs find a positive charge that increases the body's phagocytic activity and the death of cancer cells^56^. In one study, Ahamed et al. showed that CuO NPs had the genotoxic toxicity on A549 cells. These metallic NPs caused apoptosis via lipid peroxidation and oxidative stress in lung cancer cells. But, Cu ions released from the surface of CuO NPs have insufficient toxicity against the other cancer cells^57^. In 2020, Duan et al. proved that green-synthesized ZnO NPs could induce the apoptosis in A375 cancer cells by increasing the amount of ROS^4^. Besides, Dobrucka et al*.* showed that green-fabricated bimetallic ZnO/CuO NPs could stop the cell division and cell death in T98G cancer cells by inhibiting the cell cycle in the G2-M stage. In another study, Elemike et al*.* demonstrated that bimetallic ZnO/CuO synthesized by low cost and green method had less anticancer effects on Hela cancer cells than the control drug 5-fluorouracil^52^. Green synthesis of bimetallic ZnO/CuO using *Sambucus nigra *L. plant extract was illustrated, with the advantages of simplicity, cost-effectiveness, low temperature, eco-friendliness, desirable anticancer properties, and efficient cytotoxicity of biogenic ZnO/CuO NPs^51^. Using synergistic biological effects such as increased ROS production, Cu genotoxicity, and increased cancer cell apoptosis, these materials can be employed as effective therapeutic agents in the treatment of lung and melanoma cancer.

The medicinal plant black elderberry with the scientific name of *Sambucus nigra L.* from the Caprifoliaceae family was collected from the forest of Mazandaran, Iran. This plant contains different types of secondary metabolites including glycosidic compounds, terpenoids, phenolics, tannins, anthocyanins, etc. In traditional medicine, *S. nigra* was utilized to treat bone fractures, kidney diseases, respiratory diseases, and eczema^58^. Generally, green and eco-friendly synthetic strategies are deployed instead of conventional industrial manufacturing methods to eliminate/prevent hazardous materials and reduce expensive/complex instruments and materials. These eco-friendly methods have some benefits compared to the conventional physicochemical approaches such as simplicity, rapidness, cost-effectiveness, low toxicity, and environmentally friendliness. In this study, bimetallic ZnO/CuO NPs have been synthesized using *S. nigra *L. extract via a green and simple method. The synthesized bimetallic NPs have been characterized by X-ray diffraction (XRD), field emission gun scanning electron microscopy (FEG-SEM), and transmission electron microscopy (TEM) combined with energy dispersive analysis of X-rays (EDX) and selected area electron diffraction (SAED). Additionally, the anticancer activity of these NPs have been evaluated on human melanoma cell line (A375) and human lung cancer (A549).

## Materials and methods

### Materials

Dulbecco's Modified Eagle's Medium (DMEM; GIBCO), antibiotics like penicillin (GIBCO), and streptomycin (GIBCO) were purchased from Invitrogen, U.K. Fetal Calf Serum (FCS; GIBCO), glutamine^®^ (GIBCO), and sodium pyruvate (GIBCO) were purchased from Invitrogen USA. Acetic acid, zinc (II) acetate (Zn(CH_3_COO)_2_ * 2H_2_O ≤ 100%), copper (II) chloride (CuCl_2_ * 2H_2_O ≥ 99%) and sodium hydroxide (NaOH ≥ 99.0%) were purchased from Merck. 3-(4,5-Dimethylthiazol-2-yl)-2,5-diphenyltetrazolium bromide (MTT), sodium hypochlorite solution (NaOCL, available chlorine 10–15%), and dimethyl sulfoxide (DMSO) were obtained from Sigma-Aldrich.

### Green synthesis of ZnO/CuO NPs

Fresh shoots of *S. nigra* shrub were sterilized with 5% NaOCL and their surface moisture was removed at 25 °C. The plant material was dried with an electric grinder to turned into a soft powder. Then, 7 mL of deionized water was added to 1 g of plant powder, and it was shaken overnight at 25 °C. The prepared mixture was brewed at 100 °C for 10 min. Finally, the plant extract was separated by filter paper and centrifugation. To support the reproducibility, voucher specimens for the described plants were deposited in University public herbarium. The *S. nigra* Fresh shoots were collected under the licence of the collection of plant or seed specimens in the University in accordance with applicable institutional, national, and international rules and legislation. It was verified by the Iranian Botanical Survey, whose voucher specimen number was 1400/8 deposited at the Department Pharmacognosy, Kerman University.

To synthesize bimetallic NPs, 1.6 g of Zn(CH_3_COO)_2_ was added to 100 mL of extracts at 70 °C. After dissolving the zinc salt with a strainer, 0.8 g of CuCl_2_ was added to the mixture. The pH of the solution was adjusted to 8 by adding 1 mol/L of NaOH. The mixture was sterilized at 70 °C for 3 h. The ensuing NPs were washed 3 times with deionized water and dried at 90 °C in an oven. Finally, the synthesized NP powder was calcined at 400 °C for 6 h.

### Characterization of NPs

XRD analysis was performed with X'PertPro device from Panalytical Holland Company with Anod material Cu (1.54 Å, 40 kV, 30 mA) within a 2θ range of 10° to 80° in order to confirm the expected crystal structure of hexagonal ZnO and monoclinic CuO nanocrystals. The size, surface morphology, and elemental analysis of synthesized NPs were studied by FEG-SEM (microscope Sigma VP; ZEISS, Germany) and EDX analyses (EDX detector from Oxford Instruments Company; UK). The NP analysis at higher magnifications was performed by means of TEM (microscope Tecnai G2 Spirit Twin; FEI, Czech Republic). The TEM microscopy was performed at accelerating voltage of 120 kV and yielded not only standard bright-field images (TEM/BF) of the individual NPs, but also EDX spectra (TEM/EDX; EDX detector from EDAX; USA) and electron diffraction patterns (TEM/SAED; verification of crystalline structure from XRD).

### Culturing of cells

Cancer standard cell lines including human melanoma cells A375 and human lung cancer A549 have been prepared from the Pasture Institute Cells Bank, Tehran, Iran. Cancer cells were grown in Dulbecco's Modified Eagles Medium (DMEM) containing 100 IU/mL penicillin and 100 µg/mL streptomycin, 10% Fetal Calf Serum (FCS), 4 mM glutamine^®^ and 1 mM sodium pyruvate by incubating at 37 °C in a moistened atmospheric chamber and 5% CO_2_.

### Assessment of anticancer activity

The effect of bimetallic NPs was evaluated on the susceptibility of A375 and A549 cell lines using MTT assay. After 24 h of culturing cancer cells in microplates, 100 μL of the ZnO/CuO bimetallic NPs in the concentrations of 1, 10, 100, 500, and 1000 μg/mL were added to each well. Treated plates with ZnO/CuO bimetallic NPs and Doxorubicin with 4 and 8 μg/mL (as positive controls) were incubated at 37 °C and 5% CO_2_ for 72 h. Then, 20 μL of MTT solution was added to the treatments and after 4 h of incubation, 100 μL of dimethyl sulfoxide (DMSO) was added to each well. Finally, the absorbance was measured at 490 nm by an enzyme-linked immunosorbent assay (ELISA) reader (BioTeks Elx 800). The survival rate (%) was calculated by the following formula:

$${\text{Survival rate }}\left( \% \right) = \left( {{\text{OD in experimental group}}/{\text{OD in control group}}} \right) \times { 1}00$$

The inhibitory concentration needed for 50% cytotoxicity (IC_50_) was assessed using the Probit analysis and plotting the level of inhibition *versus* the concentration.

### Statistical analysis

All experiments were carried out in triplicate. Data were analyzed using ANOVA followed by unpaired Student’s *t*-test. *P*-values of < 0.05 were considered statistically significant.

P-values of < 0.01 were considered statistically significant.

### Ethical statement

We confirm that all methods were carried out under in vitro condition.

## Results

### Characterization of ZnO/CuO bimetallic NPs

#### XRD analysis

**Figure 1:**
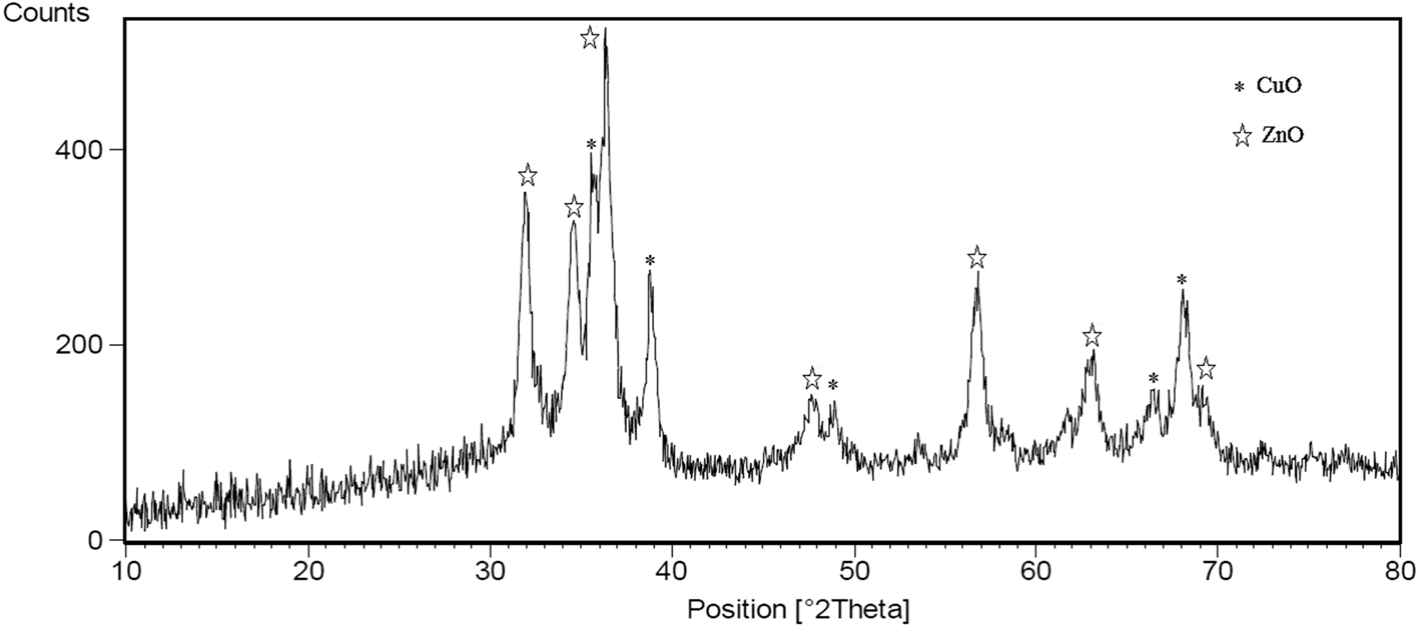
XRD spectrum of the bimetallic ZnO/CuO NPs.

The crystalline structure of the zinc-copper bimetallic NPs calcined at 400 °C is illustrated in Fig. 1. The prominent peaks observed in 2θ = 32, 34.5, 37, 47.5, 57, 63 and 69° confirmed the crystalline structure and hexagonal phase of zinc oxide NPs (JCPDS card 01-080-0075). The peaks in the 2θ = 36, 39, 49, 66.5 and 68.5° confirmed the crystal structure and monoclinic copper oxide phase (JCPDS card 01-080-1917)^59^. As can be observed in the Fig. 1, the number of clear diffraction and peak intensities of ZnO NPs are higher than of CuO NPs. This indicates that ZnO NPs have higher percentage in the structure of zinc-copper bimetallic NPs and high degree of crystallinity. The peak of low crystallization of CuO NPs is due to the coating role of ZnO NPs on them^60^.

#### SEM analysis

**Figure 2:**
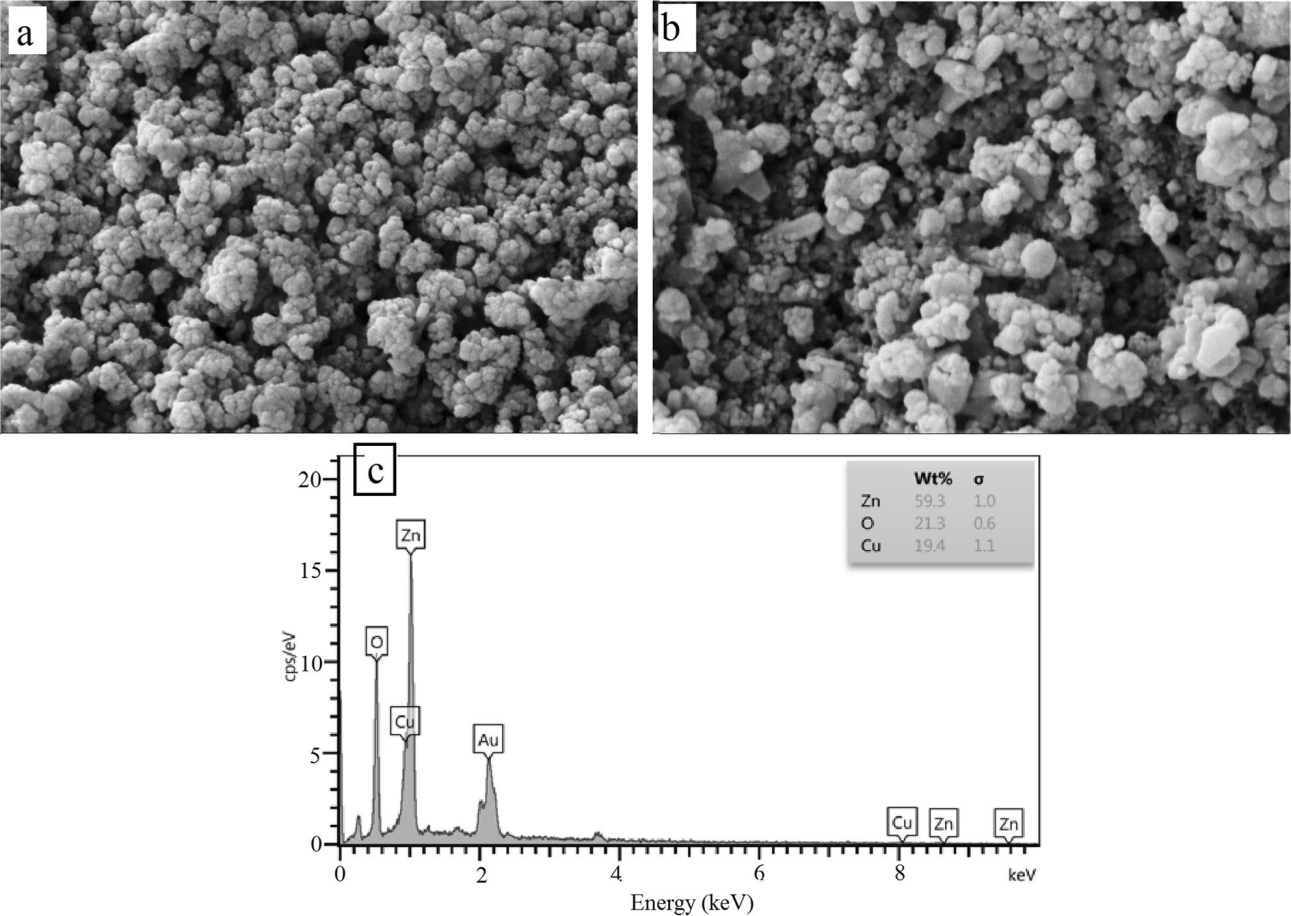
SEM images (**a, b**) and EDX spectra (**c**) of the ZnO/CuO NPs.

The surface morphologies, particle sizes and EDX measurements of the bimetallic NPs are shown in Fig. 2. Consequently, Fig. 2a,b depicts that a number of polygonal ZnO NPs^61^ are arranged in adjacent spherical NPs. The growth of polygonal shapes of NPs was appropriate in different directions. Figure 2b shows isometric NPs, where size ranged from 20 to 130 nm due to the different shapes of ZnO NPs with CuO. In some portions, the diameter of the prepared ZnO NPs was 98 nm, and also the diameter of some aggregated spherical particles was 130 nm. The presence of Zn, Cu, and O with weight percentages of 59.3, 19.4, and 21.3, respectively, confirmed the high amount of ZnO NPs compared to CuO (Fig. 2c).

#### TEM analysis

**Figure 3:**
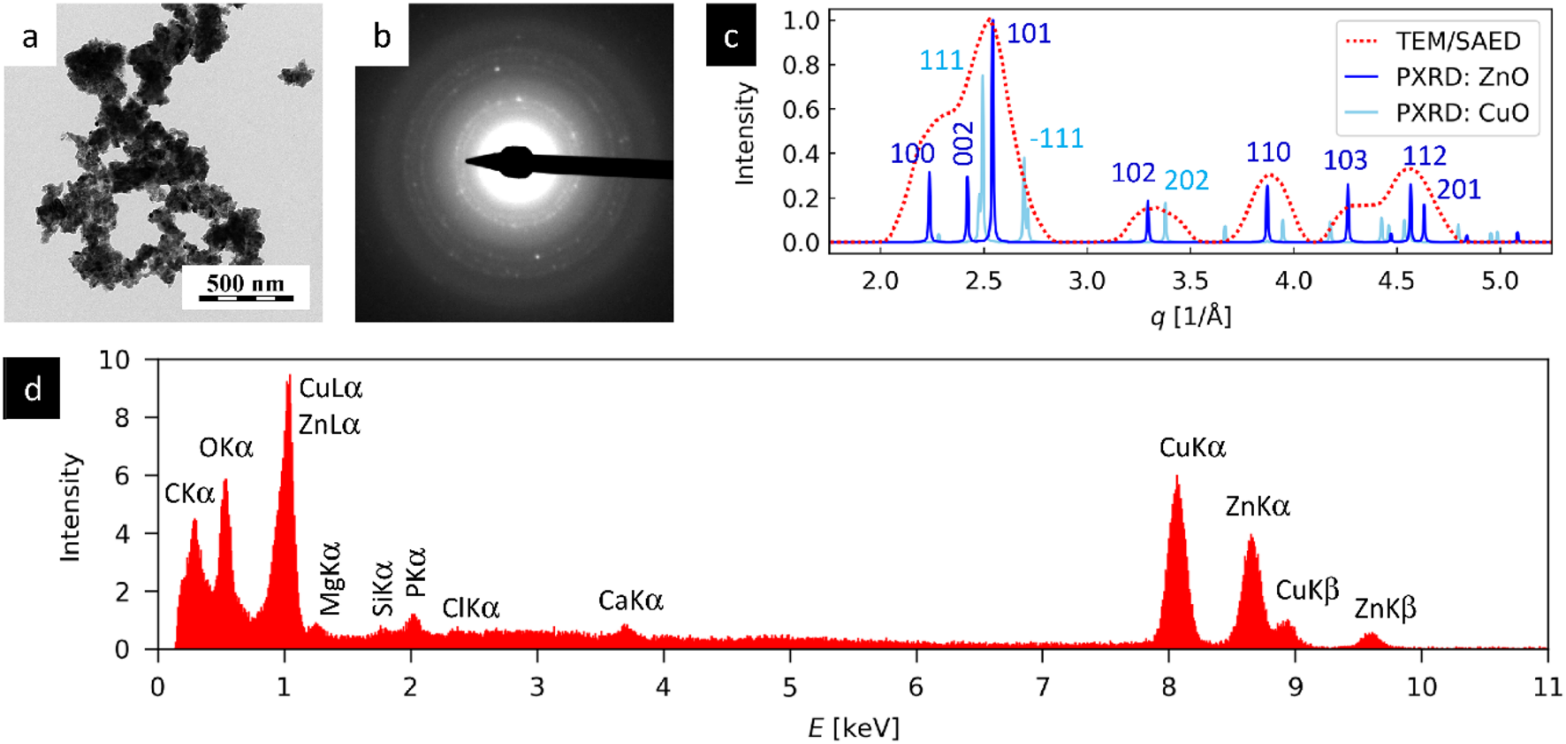
TEM analysis of ZnO/CuO NPs: (**a**) TEM/BF image demonstrates the morphology of NPs, (**b**) TEM/SAED diffraction pattern, (**c**) the comparison of radially averaged experimental TEM/SAED diffractogram with theoretically calculated power XRD diffractograms of ZnO and CuO nanocrystals, and (**d**) TEM/EDX spectrum of bimetallic ZnO/CuO NPs.

The detailed analysis of ZnO/CuO bimetallic NPs by TEM (Fig. 3) showed the NPs in thin layer deposited on carbon film (TEM/BF; Fig. 3a), confirming their crystalline structures (TEM/SAED; Fig. 3b,c) and their elemental composition (TEM/EDX; Fig. 3d). The TEM/BF micrograph showed that ZnO/CuO bimetallic NPs were mostly isometric, tended to form agglomerates during the drying process, and exhibited quite broad particle size distribution (particle diameters from tens to hundreds of nm). The TEM/SAED diffractogram (Fig. 3b) exhibited quite low diffraction intensities. This could be attributed to the fact that relatively low concentration of small particles was deposited on carbon film and the nanoparticle diffractions were hidden in amorphous carbon background. After careful background correction, the comparison of radially averaged TEM/SAED diffraction pattern with theoretically calculated powder XRD of hexagonal ZnO and monoclinic CuO nanocrystals showed quite good agreement (Fig. 3c), confirming the expected crystalline structure. The processing of TEM/SAED patterns and calculation of theoretical power XRD patterns was described in our previous studies^30,31^. The TEM/EDX spectrum was dominated by peaks of Cu (~ 11%), Zn (~ 8%), O (~ 25%) and C (~ 37%), while the concentrations of all other elements (Mg, Ca, Cl, Si and P) were below 1%. The high concentration of carbon was due to the fact that the NPs were deposited on the supporting carbon film. The presence of Cu, Zn, and O in approximate ratio 1:1:2 corresponded to the expected composition of ZnO/CuO NPs. The low concentrations of other elements indicated that a small amount of compounds from NP synthesis was remained in the solution and deposited on the carbon film together with the NPs.

### Cytotoxicity of the NPs

**Figure 4:**
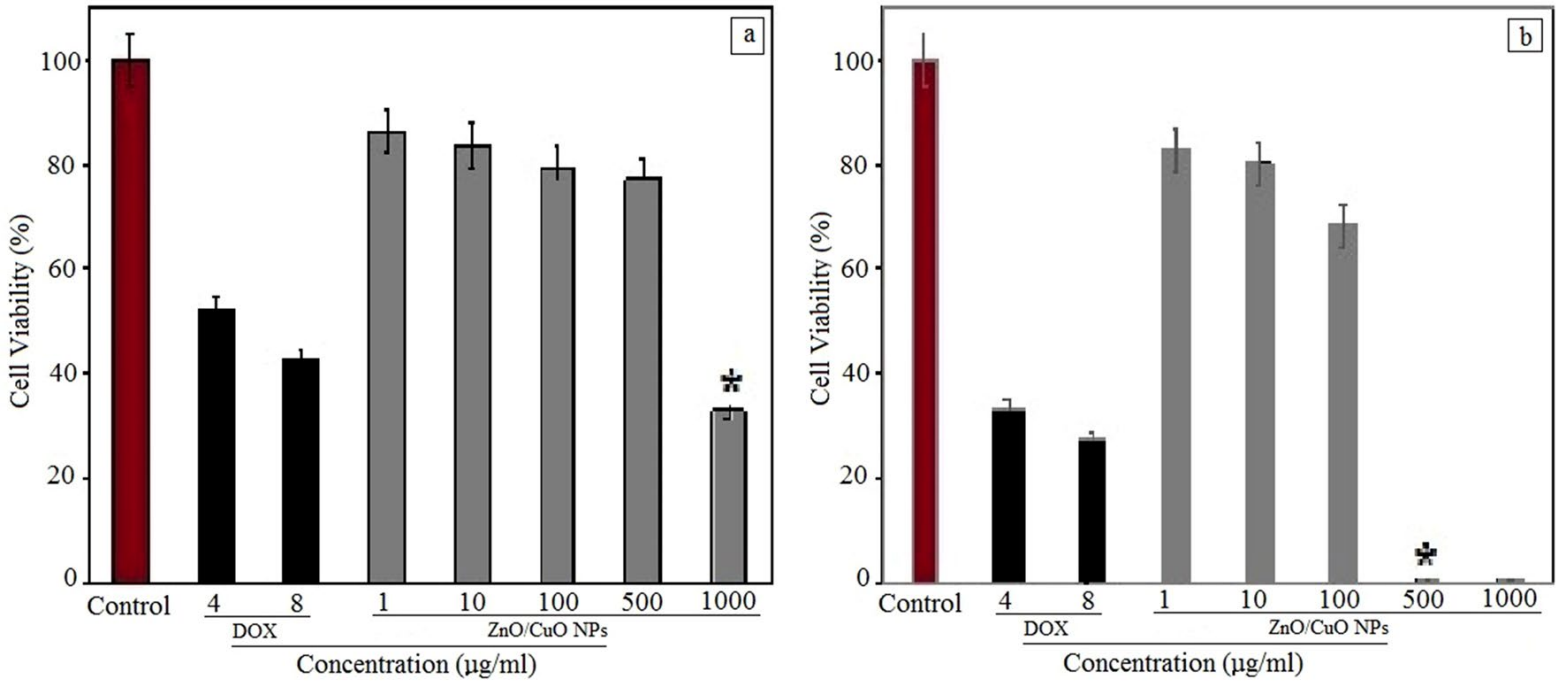
The cell viability (in %) of (**a**) A549 cell line (Human lung cancer cells) and (**b**) A375 cell line (human melanoma cancer cells) in the presence of various concentrations of ZnO/CuO NPs, doxorubicin (positive control) and untreated cells (negative control). *P < 0.01.

The in vitro cytotoxic effects of bimetallic ZnO/CuO NPs on A375 and A549 are shown in Fig. 4. The cytotoxicity of untreated cells and doxorubicin (4 and 8 µg/mL) was evaluated as negative and positive control, respectively. The index of anti-proliferative activity of ZnO/CuO bimetallic NPs on A375 cells IC_50_ was 41 µg/mL while this value for A549 cells was 785 µg/mL. Figure 4 shows that these NPs are not very toxic against A549 cancer cells while their toxicity is higher than the evaluated positive control (doxorubicin). These NPs showed remarkable cytotoxic effects towards A375 cancer cells, and at a concentration of 500 µg/mL, the number of cancer cells has reached zero (Fig. 4).

## Discussion

The potential therapeutic effects of trace elements against cancers made the metal and metal oxide nanomaterials promising candidates for cancer treatment and diagnosis. Trace elements in the body such as zinc (Zn), copper (Cu), iron, iodine, fluoride, chromium, selenium, manganese and molybdenum are involved in the structure of metalloenzymes, proteins, immune function DNA production, enzyme function, hormones and antioxidant function. Absorption and accumulation of Cu by cancer cells can cause the angiogenesis of tumors, tumor growth, and proliferation. Since, there is a competition in the uptake of Cu and Zn in cells, the serum levels of them have been evaluated in a variety of cancers^62,63^. Interestingly, changes in serum levels of Cu to Zn play vital roles in the diagnosis and progression of lung cancer; therefore, the increasing serum Cu levels to Zn has led to the spread of malignant lung tumors^62^. However, serum Cu levels in patients with melanoma are not varied from those in healthy individuals^64^. In this study, the bimetallic ZnO/CuO NPs were successfully synthesized in one-step using *S. nigra* extract. Therefore, we showed the promising potentials of this plant extract for the green, eco-friendly, and cost-effective fabrication of ZnO/CuO NPs. The synthesis of NPs using conventional physico-chemical methods may include the application of expensive devices.materials, toxic/hazardous precursors, high temperatures and pressures, and complicated steps^60^.

The formation of NPs were confirmed by XRD, FEG-SEM, and TEM. The synthesized NPs had hexagonal and monoclinic crystalline structures. The presence of Zn, Cu, and O ions in NPs were verified by EDX analysis. The MTT assay shows the anti-cancer activity of these bimetallic NPs against cancer A549 and A375 cancer cells. Notably, the high toxicity of positive control on A549 cancer cells compared to the prepared ZnO/CuO NPs can be related to the presence of Cu in the synthesized bimetallic ZnO/CuO NPs. However, the bimetallic NPs had a favorable effect on the mortality of A375 cancer cells. According to Zarghami et al*.*^65^, there was a direct relationship between Cu levels and telomerase gene expression in A549 cancer cells. The enzyme telomerase is active in all cancer cells, and the level of telomerase activity is directly related to the progression of A549 cells. With increase in amount of Cu, the higher activity of this enzyme is occurred, which can result in the development and proliferation of the lung tumors. However, Zn levels do not play a critical role in the progression of this disease^65^. Also, these green-synthesized NPs can be employed for the diagnosis and treatment of other cancer cell lines.

## Conclusions

A simple and inexpensive method was established for the green and eco-friendly synthesis of bimetallic ZnO/CuO NPs using *S. nigra* extract as a reducing and stabilizing agent. The high purity of these bimetallic NPs as well as the monoclinic and hexagonal crystalline structures of ZnO and CuO NPs were confirmed by XRD and TEM/SAED diffractograms. The ZnO and CuO NPs with different shapes were existed in the product where the diameter of these NPs ranged approximately from 20 to 130 nm. These bimetallic NPs exhibited low toxicity on A549 cancer cells while their toxicity on A375 cancer cells was dose-dependent and the survival of cancer cells was reduced via an increase in the concentration of the bimetallic NPs. It can be concluded that the serum level of trace elements in different cancer cells is different, and according to the serum level of each element, an appropriate treatment strategy can be deployed for the relevant cancer.
